# Supplementary material for: Causal Relationship between Aging and Anorexia Nervosa: A White-Matter-Microstructure-Mediated Mendelian Randomization Analysis
Source: Biomedicines. 2024 Aug 16;12(8):1874. doi: 10.3390/biomedicines12081874 (PMC11351342; doi:10.3390/biomedicines12081874)
Supplement: Supplementary file 1 [file biomedicines-12-01874-s001.zip › Supplementary Figure/Figure S4/Singe SNP plot of MR analysis for TL to ubm-b-1769.pdf]

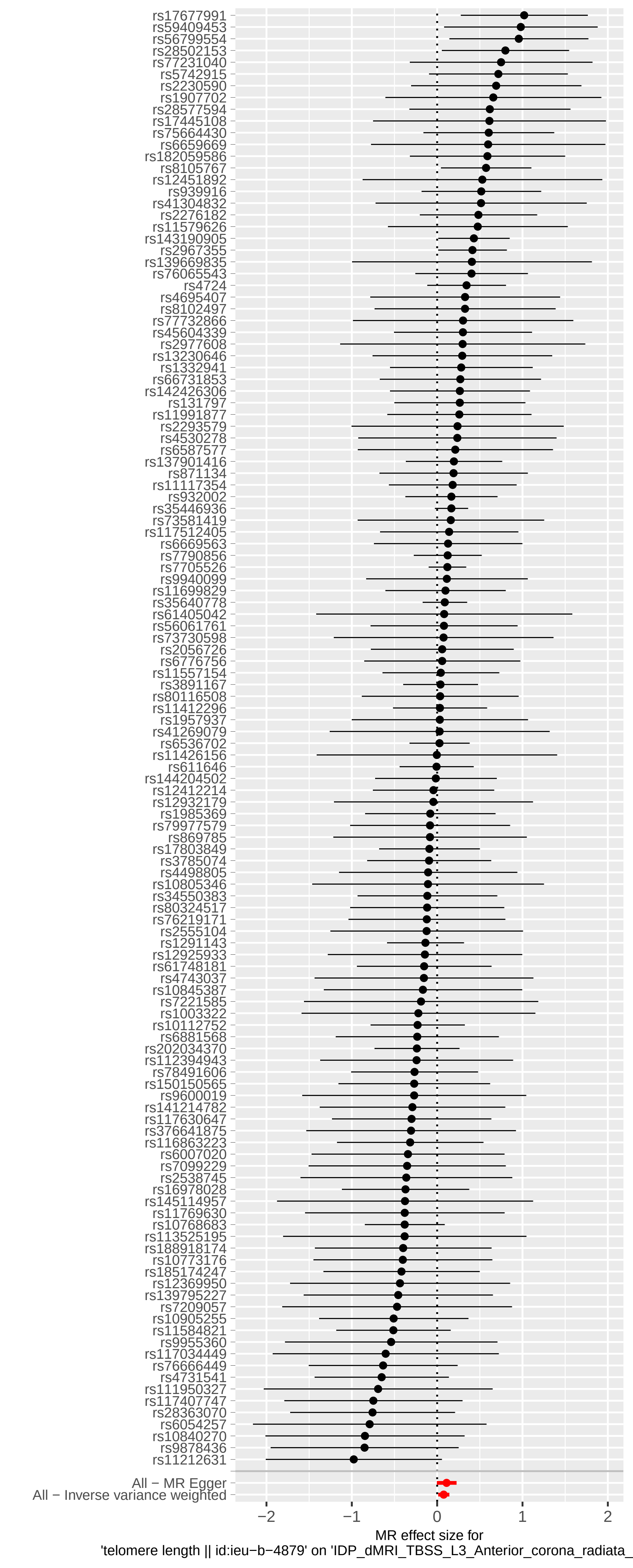
rs17677991
rs59409453
rs56799554
rs28502153
rs77231040
rs5742915
rs2230590
rs1907702
rs28577594
rs17445108
rs75664430
rs6659669
rs182059586
rs8105767
rs12451892
rs939916
rs41304832
rs2276182
rs11579626
rs143190905
rs2967355
rs139669835
rs76065543
rs4724
rs4695407
rs8102497
rs77732866
rs45604339
rs2977608
rs13230646
rs1332941
rs66731853
rs142426306
rs131797
rs11991877
rs2293579
rs4530278
rs6587577
rs137901416
rs871134
rs11117354
rs932002
rs35446936
rs73581419
rs117512405
rs6669563
rs7790856
rs7705526
rs9940099
rs11699829
rs35640778
rs61405042
rs56061761
rs73730598
rs2056726
rs6776756
rs11557154
rs3891167
rs80116508
rs11412296
rs1957937
rs41269079
rs6536702
rs11426156
rs611646
rs144204502
rs12412214
rs12932179
rs1985369
rs79977579
rs869785
rs17803849
rs3785074
rs4498805
rs10805346
rs34550383
rs80324517
rs76219171
rs2555104
rs1291143
rs12925933
rs61748181
rs4743037
rs10845387
rs7221585
rs1003322
rs10112752
rs6881568
rs202034370
rs112394943
rs78491606
rs150150565
rs9600019
rs141214782
rs117630647
rs376641875
rs116863223
rs6007020
rs7099229
rs2538745
rs16978028
rs145114957
rs11769630
rs10768683
rs113525195
rs188918174
rs10773176
rs185174247
rs12369950
rs139795227
rs7209057
rs10905255
rs11584821
rs9955360
rs117034449
rs76666449
rs4731541
rs111950327
rs117407747
rs28363070
rs6054257
rs10840270
rs9878436
rs11212631
All − MR Egger
All − Inverse variance weighted
−2
−1
0
1
2
MR effect size for
'telomere length || id:ieu−b−4879' on 'IDP_dMRI_TBSS_L3_Anterior_corona_radiata_
